# Mortality and causes of death among Croatian male Olympic medalists

**DOI:** 10.3325/cmj.2017.58.263

**Published:** 2017-08

**Authors:** Vedran Radonić, Damir Kozmar, Darko Počanić, Helena Jerkić, Ivan Bohaček, Tomislav Letilović

**Affiliations:** 1 Institute for Emergency Medicine Sisak and Moslavina County, Sisak, Croatia; 2 Department of Cardiology, Zagreb University Hospital Merkur, Zagreb, Croatia; 3 Department of Orthopaedic Surgery, Zagreb University Hospital Center and School of Medicine, Zagreb, Croatia; 4 Department of Cardiology, Zagreb University Hospital Merkur and Zagreb University School of Medicine, Zagreb, Croatia

## Abstract

**Aim:**

To compare the overall and disease-specific mortality of Croatian male athletes who won one or more Olympic medals representing Yugoslavia from 1948 to 1988 or Croatia from 1992 to 2016, and the general Croatian male population standardized by age and time period.

**Methods:**

All 233 Croatian male Olympic medalists were included in the study. Information on life duration and cause of death for the Olympic medalists who died before January 1, 2017, was acquired from their families and acquaintances. We asked the families and acquaintances to present medical documentation for the deceased. Data about the overall and disease-specific mortality of the Croatian male population standardized by age and time period were obtained from the Croatian Bureau of Statistics (CBS). Overall and disease-specific standard mortality ratios (SMR) with 95% confidence intervals (CI) were calculated to compare the mortality rates of athletes and general population.

**Results:**

Among 233 Olympic medalists, 57 died before the study endpoint. The main causes of death were cardiovascular diseases (33.3%), neoplasms (26.3%), and external causes (17.6%). The overall mortality of the Olympic medalists was significantly lower than that of general population (SMR 0.73, 95% CI 0.56-0.94, *P* = 0.013). Regarding specific causes of death, athletes’ mortality from cardiovascular diseases was significantly reduced (SMR 0.61, 95% CI 0.38-0.93, *P* = 0.021).

**Conclusions:**

Croatian male Olympic medalists benefit from lower overall and cardiovascular mortality rates in comparison to the general Croatian male population.

Regular physical activity improves general health and reduces the risk of premature mortality (1,2). People who exercise regularly have a lower risk of adverse cardiovascular events (3). In addition, exercise decreases oxidative stress and inflammation, preventing chronic diseases (4). A sport-oriented lifestyle involves regular sleep, balanced nutrition, and the avoidance of unhealthy habits, such as smoking, alcohol, and drugs. Obesity, type 2 diabetes, arterial hypertension, and ischemic heart disease are more common in people with sedentary lifestyles and they are associated with shorter life expectancy (5,6).

Challenges in sport drive the human body to develop better coordination, increased muscle strength, higher aerobic and anaerobic capacities, and better cognitive skills (7). At the same time, elite athletes are exposed to strenuous training, which may have negative effects on their health, such as potentially harmful effects of vigorous exercise on the cardiovascular system (8). Furthermore, at the top level of competition, athletes often deal with severe stress due to pressure and fear of failure. Strenuous training regimes and an extreme lifestyle often disrupt their social life and education from early youth (9). Despite prospective fame and success, the long-term risk of anxiety and depression is not to be overlooked (10). Still, a recent study showed that vigorous exercise may provide greater health benefits compared to moderate exercise (11).

Among all the athletes, Olympic medalists are the very best and as such, they are exposed to extreme training conditions. According to the largest retrospective study including 15 174 Olympic medalists from nine country groups (United States of America, Germany, Nordic countries, Russia, United Kingdom, France, Italy, Canada, and Australia and New Zealand), Olympic medalists live longer than their fellow citizens; however, that study did not examine the causes of death of the athletes (12).

Elite athletes show variations in lifestyle, training regime, and doping abuse depending on the country where they live (13). Populations of different countries significantly differ in living conditions, which result in variations of mortality rates. For example, Croatia has higher mortality rates in comparison with the European Union rates in all three leading causes of death groups: cardiovascular, neoplasms, and the external causes (14). Some retrospective cohort studies of elite athletes compared overall and disease-specific mortality of the athletes and corresponding general population (15-20). These studies together improve the knowledge on the influence of elite sport on health and mortality outcomes.

The aim of this retrospective cohort study was to examine the overall and disease-specific mortality among Croatian male Olympic medalists in comparison with the general Croatian male population.

## PARTICIPANTS AND METHODS

### Data collection

This a retrospective cohort study of male Croatian Olympic medalists' mortality and causes of death. The cohort included all Croatian male athletes who won one or more Olympic medals representing Yugoslavia from 1948 to 1988 and Croatia from 1992 to 2016 (21). Croatian female Olympic medalists were not included in this study, because only 23 Croatian women won Olympic medals in the period from 1968 to 2016 and all of them were alive at the study endpoint (21).

We collected data on the life duration and cause of death in the Olympic medalists who died before January 1, 2017, from their families and acquaintances. We asked the families and acquaintances to present medical documentation for the deceased. Data on age-specific, disease-specific, and period-specific mortality among the male population living in what is today the Republic of Croatia from 1948 to 2016 was collected from the Croatian Bureau of Statistics (CBS). We traced athletes from the first day of the year they won their first Olympic medal to the day of their death (if applicable) or study endpoint, whichever came first. Follow-up was measured in years.

### Statistical analysis

To compare the overall, disease-specific, and age group mortality rates in Croatian male Olympic medal winners and corresponding general population, we used the standard mortality ratio (SMR) with 95% confidence interval (CI). The SMR calculation was performed by dividing the number of deaths in the observed cohort with the number of expected deaths in the general population standardized by age and time period (22).

Athletes were divided by age in ten-year groups starting with a group aged 15-24 years. The Croatian general population statistics came from decennial censuses, starting in 1951. Where the specific age group data were insufficient, we used the nearest available census estimate statistics. Death rates for all the age groups in corresponding general population in a single year multiplied by the number of athletes in a particular age group provided the number of expected deaths for that year in the general population. The total number of expected deaths among the Croatian male population was calculated as the sum of expected deaths for every year during the observed period. The same method was used to calculate the number of disease-specific and age-specific expected deaths in the Croatian male population. For calculating the overall, disease-specific, and age group-specific SMRs with 95% CI, we used the Mid-P exact test with Miettinen’s modification (23). Results were considered statistically significant if the *P* value was less than 0.05. Observed deaths in our cohort and expected deaths in the general population during the follow-up time are presented as Kaplan-Meier curves. Microsoft Office Excel 2013 (Redmond, WA, USA) software was used for the statistical analysis.

## RESULTS

### Overall mortality

From 1948 to 2017, a total of 233 Croatian male athletes won at least one Olympic medal including 73 water polo players, 49 handball players, 36 soccer players, 29 basketball players, 20 rowers, 4 tennis players, 4 boxers, 3 wrestlers, 3 sailors, 2 kayakers, 2 archers, 1 swimmer, 1 weightlifter, 1 hammer thrower, 1 marathoner, 1 alpine skier, 1 table tennis player, 1 gymnast, and 1 biathlete (14). Among them, 57 athletes died before the study endpoint including 27 soccer players, 15 water polo players, 4 handball players, 4 rowers, 2 basketball players, 2 kayakers, 1 hammer thrower, 1 marathoner, and 1 boxer.

**Figure 1:**
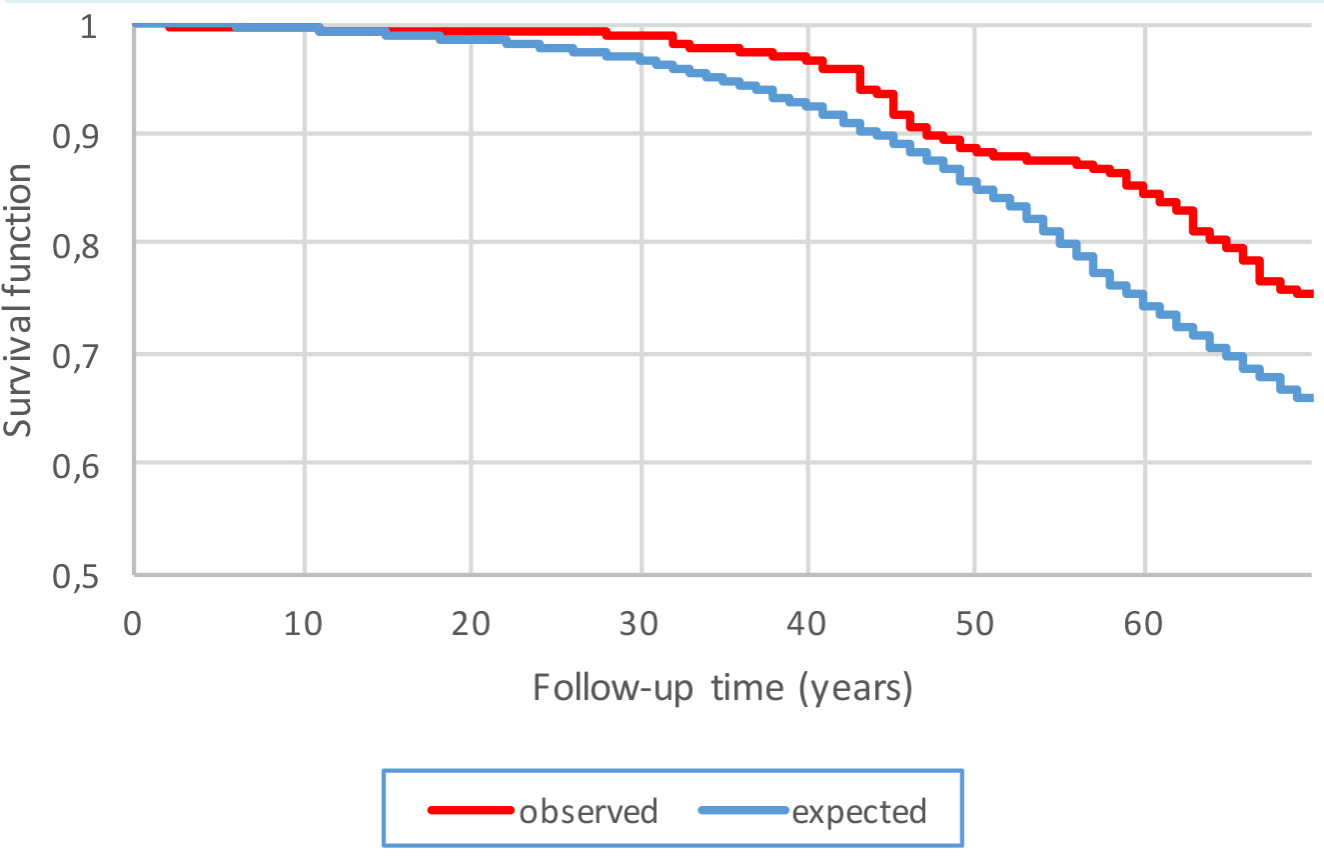
Kaplan-Meier curve representing Croatian male Olympic medal winners’ survival (red) compared to the survival of the Croatian general male population (blue).

Of a total of 233 Croatian male Olympic medal winners included in the study cohort, 57 deceased in the observed period. The participants' mean age of entering the study was 25.2 ± 3.6 years and the mean follow-up time of participants was 29.1 ± 17.7 years (a total of 6782.5 person years). Overall SMR was 0.73 (95% CI, 0.56-0.94, *P* = 0.013). The mean life duration of the deceased Olympic medalists was 67.7 ± 16.3 years. The youngest athlete passed away at the age of 29 and the oldest at the age of 95. The death rate among the Olympic medalists over time was lower than expected (Figure 1).

### Causes of death

The main causes of death of the athletes were cardiovascular diseases, followed by neoplasms and external causes, such as traffic accidents, falls, and assaults (Table 1). The rest of the causes of death in 13 Olympic medalists, including musculoskeletal diseases, infectious diseases, mental disorders, nervous system diseases, endocrine, nutritional and metabolic diseases and unclear causes of death, were classified as other causes of death.

**Table 1:** Croatian male Olympic medal winners’ and Croatian general population’s standard mortality ratio per causes of death and overall mortality*

Cause of death	No. (%) of deaths observed	Death expected	SMR (95% CI)	*P*
Cardiovascular	19 (33.3)	31.18 (40.0)	0.61 (0.38-0.93)	0.021
Neoplasms	15 (26.3)	21.58 (27.7)	0.70 (0.40-1.12)	0.147
External	10 (17.6)	8.11 (10.4)	1.23 (0.63-2.20)	0.492
Other	13 (22.8)	17.12 (21.9)	0.76 (0.42-1.27)	0.322
Overall	57 (100.0)	77.97 (100.0)	0.73 (0.56-0.94)	0.013

*Abbreviations: SMR – standard mortality ratio, CI – confidence interval.

The overall number of deaths was found to be lower in the observed cohort of Olympic medalists than in the general population, as well as the group of cardiovascular causes (Table 1). Although the number of deaths due to external causes was higher among the athletes, the difference was not statistically significant.

### Mortality by age groups

We found a significantly lower mortality rate among Olympic medalists in the 45-54 and 65-74 age groups (Table 2). In the 25-34, 35-44, 55-64, and >75 age groups, athletes’ mortality did not differ significantly from the mortality in general population. The mortality rate for the 15-24 age group was not analyzed because no athletes died in that age group.

**Table 2:** Croatian male Olympic medal winners’ and the Croatian general population’s standard mortality ratio per age groups*

Age group (years)	No. of deaths observed	Death expected	SMR (95% CI)	*P*
25-34	2	3.09	0.64 (0.10-2.13)	0.589
35-44	3	6.07	0.49 (0.12-1.34)	0.204
45-54	4	10.78	0.37 (0.11-0.89)	0.023
55-64	16	16.11	0.99 (0.58-1.57)	0.999
65-74	9	21.20	0.42 (0.20-0.77)	0.003
>75	23	20.32	1.13 (0.73-1.67)	0.538

*Abbreviations: SMR – standard mortality ratio, CI – confidence interval.

## DISCUSSION

To the best of our knowledge, this is the largest retrospective cohort study about mortality and cause of death among Croatian male elite athletes. Our results showed that Croatian male Olympic medal winners had 27% lower mortality rate in comparison with the general Croatian male population. This result corresponds to previously published findings on lower overall mortality in French Olympians, French Olympic rowers, and Polish Olympians by 49%, 42%, and 50%, respectively, while a large multinational study of Olympic medal winners reported 8% survival advantage of the athletes 30 years after winning their first Olympic medal compared to the matched cohort in the general population (12,15,16,24).

In our study, mortality was significantly lower in the 45-54 and 65-74 age groups. Among the athletes younger than 45, the lack of difference in mortality may be explained by the relatively small number of events (deaths), both observed and expected. In the 55-64 and >75 age groups, athletes’ mortality was not found to be significantly lower. No difference in the oldest age group was expected, as reported in previous literature findings (15,25). However, the lack of difference in the 55-64 age group is an unexpected finding and may be explained by the fact that some of the athletes abandoned their athletic lifestyles after their sports careers. We did not collect data on the lifestyles of the observed athletes to be able to verify this explanation.

The Kaplan-Meier survival curves showed the difference between the observed and expected mortality rates during the follow-up. Athletes’ survival was higher than that of the general population during the entire follow-up, but the level of difference varied. From 1968 to 1987 (20 to 39 years of follow-up), the difference increased. From 1988 to 1995 (40 to 47 years of follow-up), the difference became smaller because more deaths were observed than expected in that period, mostly among the athletes in the 55-64 age group. At that time, the Homeland War was fought in Croatia in from 1991 to 1995. Although no Croatian Olympic medal winners’ death occurred as a direct consequence of the war, we still cannot rule out the possible indirect connection of the deaths with the war and all the associated events. It could explain the convergence of the survival curves observed in this period. After 1995, the difference increased again and remained relatively constant when a lot of athletes reach old age. We compared our results with those from the similar study of French Olympians who were also categorized in age groups (15). Our survival curves results and theirs were not entirely concordant; they found lower athletes’ mortality in all age groups except the oldest one, and the difference between their observed and expected mortality waned during the follow-up (15). The difference between our study and the French study may be explained by variance in lifestyle, living conditions, and training program among the athletes from different countries (26). However, no data was collected with the potential to prove these assumptions.

In our study, the survival advantage of Croatian Olympic medalists seems to be associated with 39% lower cardiovascular mortality compared with the general population. Cardiovascular mortality was also found to be lower in French Olympians, Finnish elite athletes, Italian soccer players, American football players, French Olympic rowers, and French Tour de France participants (15-20). The positive effects of moderate physical activity on cardiovascular health are well described in the literature (3). The American College of Cardiology and the American Heart Association recommend 3 to 4 sessions per week of moderate to vigorous physical activity, lasting on average 40 minutes per session (1). Still, the possibility of strenuous training having some harmful effects on cardiovascular health is still under debate. Some studies found it connected with atrial fibrillation, myocardial fibrosis, artery calcification, and myocardial injuries with high troponin level, whereas other studies did not find these connections (8,27-33). Despite the negative conclusions on the effects of strenuous training, a large study conducted in Taiwan showed an additional reduction of cardiovascular and all-cause mortality rate in people who regularly engage in vigorous exercise compared to those who only engage in a moderate level of exercise (11). Our results also suggest that the association between cardiovascular risk and training level may follow an L-shaped curve rather than the U-shaped curve as suggested by others (8,15).

We found neoplasms and external causes to be the second and third leading causes of death, respectively, in our cohort. The number of deaths from both causes did not significantly differ from that expected in the general population. Similar studies of elite athletes with larger cohort sample found significantly lower mortality rates from both causes for the athletes compared to the corresponding general populations (15,19).

One of the main limitations of our study is a relatively small number of participants, even though all Croatian male Olympic medalists were included in the study. It would be interesting to see if there were any differences in mortality between groups of athletes from different sports categories, such as endurance, team, and power sports, since these differences have been reported in the literature (34). Due to our relatively small sample with uneven distribution of medal winners according to the sports category, such analysis would have been of questionable value and low statistical power. Hence, such an analysis was not performed. Unlike some other countries, Croatia does not have an epidemiologic register for causes of deaths that occur in Croatian territory. In similar studies of elite athletes in other countries, these national registers were the main sources for the data collection (15,16,19,20). Because this option was not available to us, we collected data about the vital status and causes of death of the athletes from families and acquaintances and asked them to present the medical documentation for the deceased athletes.

One of the possible confounders that may have affected the study results is variation in socio-economic status. A recently published study reported that better socio-economic status is associated with greater life expectancy (35). Despite the lack of information about the earnings of the observed cohort, we can assume that the athletes may have been more privileged socio-economically compared with the general population.

Anabolic agents may decrease a life expectancy of the athletes who abuse it (36). Because no Croatian Olympic medalist ever had to return his Olympic medal due to the positive doping test, our study participants are officially considered clean of forbidden substances in the time of winning the Olympic medal. However, International Olympic Committee did not provide official doping controls until 1968 and there were no official tests for anabolic steroids at the Olympic games until 1976 (37,38).

Studies found that elite athletes smoke less than the general population and it is connected with their lower tobacco-related cancers and cardiovascular mortality (17). We did not collect data on the smoking status of our cohort.

Genetic selection bias is also not to be overlooked (29,39). All athletes were considered healthy and fit at the study entry point, but the reference group consisting of the general population included individuals suffering chronic health and disability issues. Due to the facts mentioned above, the results of our study should be interpreted with caution.

In conclusion, Croatian male Olympic medal winners have a lower overall mortality rate compared to the general male population of Croatia. The mortality rate for cardiovascular diseases, the main cause of death among the observed athletes, is significantly reduced in comparison to the general population. Further studies with larger samples and a higher level of caution for confounding factors are needed for better understanding of altered mortality rates among elite athletes.
